# A State of Alcohol Hangover Impedes Everyday Prospective Memory

**DOI:** 10.3389/fnhum.2018.00348

**Published:** 2018-09-19

**Authors:** Thomas Heffernan

**Affiliations:** Department of Psychology, Faculty of Health and Life Sciences, Northumbria University, Newcastle upon Tyne, United Kingdom

**Keywords:** alcohol hangover, executive function, prospective memory, young adults, cognition

## Abstract

The aim of the current study was to investigate what impact a state of alcohol hangover (AH) has upon everyday prospective memory (PM; memory for future events/intentions). Previous research has shown that the AH has a detrimental effect upon cognitive abilities, including memory and attentional deficits. No published research articles to date have focused upon what impact AH might have upon everyday memory, of which PM is a good example. The current study compared an AH group (AHG) with a non-hangover group (NHG) on PM. Since other drug use, anxiety and depression can affect PM independent of the AH, these covariates were controlled for in the study. Fifty-eight young adults studying at university participated in this between-subjects design study-25 in the AHG and 33 in the NHG. The Prospective Remembering Video Procedure (PRVP) measured PM. The Acute Hangover Rating Scale confirmed a state of AH and a Digital Breath Analyzer Test measured their BAC. The Hospital Anxiety and Depression Scale gauged levels of anxiety and depression and a Recreational Drug Use Questionnaire (RDUQ) measured alcohol and other drug use. Anyone who reported having used an illicit substance (e.g., cannabis, ecstasy) or who smoked, were excluded from the study. After controlling for age, alcohol units per week, years spent drinking alcohol, anxiety and depression scores, a one-way analysis of covariance (ANCOVA) revealed that the AHG (mean = 5.16) recalled significantly fewer items on the PRVP than the NHG (mean = 7.51)—*F*_(1,52)_ = 5.69, *p* < 0.05. Overall, it appeared that a state of AH significantly impaired PM, which was not attributable to age, alcohol use, or anxiety or depression indices. Given the importance of PM to everyday activities, such as remembering to keep appointments or to take an important medication on time, this finding may have farther-reaching implications. These findings should also be used to educate young adults and health professionals dealing with the consequences with regards the dangers of alcohol misuse.

## Introduction

The alcohol hangover (AH) can be defined as the presence of a combination of both mental and physical symptoms experienced the day after a single episode of heavy drinking (drinking in excess of four alcoholic drinks for women and in excess of five drinks for men), which begin when the blood alcohol concentration approaches zero (van Schrojenstein Lantman et al., 2016). These symptoms can last in excess of 24 h and include headache, drowsiness, concentration problems, dry mouth, dizziness, gastro-intestinal complaints, sweating, nausea, hyper-excitability and anxiety (Swift and Davidson, 1998; Wiese et al., 2000).

Previous research on the cognitive consequences of the AH have been dogged by methodological limitations, including the introduction of expectancy effects (where the participants’ were aware that hangover-induced cognitive impairments were expected, which may have confounded the results) and the lack of adequate (e.g., non-hangover) comparison groups (see e.g., Stephens et al., 2008; Verster, 2008—for discussion). Despite this, there is some consistency between laboratory-based studies and naturalistic studies of the AH, which have found hangover-related impairments in memory, attention and psychomotor performance (Verster et al., 2003; McKinney and Coyle, 2004; Kruisselbrink et al., 2006). More recently, behavioral studies have demonstrated that a state of AH leads to performance deficits such as slower response times and increases in response errors (see, Stephens et al., 2008; Verster, 2008; Ling et al., 2010), as well as working memory deficits (Owen et al., 2013). For example, Kim et al. (2003) found that participants in a state of AH displayed a range of cognitive deficits, including attentional and memory deficits, as well as problems in information processing, compared with non-hangover controls. In addition, the AH has also been shown to produce deficits in sustained attention and attentional selection (e.g., Rohsenow et al., 2010; McKinney et al., 2012), slower choice reaction times (Grange et al., 2016), reduced short-term recall for digit and visual patterns (Howland et al., 2010), as well as impairments in spatial and numeric working memory and inhibition (Owen et al., 2013).

It is clear that the majority of previous research has tended to focus on laboratory and/or field tests of retrospective memory function—that is the learning, consolidation, retention and retrieval of previously presented target material, with no published research articles on what impact the AH might have upon everyday cognition—of which *prospective memory (PM)* is a good example. PM refers to the cognitive ability to remember to carry out a planned action or intention at some future point in time (Brandimonte et al., 1996). PM represents an important aspect of day-to-day memory and examples of this type of memory include remembering to pay a bill by its due date, to post a letter on time or remembering to take an important medication on time. The present study aims to extend our knowledge of what impact a naturalistic state of AH has upon PM in young adults. This was achieved by comparing an AH group (AHG) and a non-hangover group (NHG) on an objective measure of PM in the form of the Prospective Remembering Video Procedure (PRVP), a measure that has been used successfully in previous drug research to determine a variety of drug-related impairments in PM (see e.g., Bartholomew et al., 2010; Heffernan et al., 2010). Since other drug use (Bartholomew et al., 2010; Heffernan et al., 2010; Parrott et al., 2002) and anxiety and depression (Schnitzspahn et al., 2014) can affect PM independent of a state of AH these were controlled for in the study.

## Materials and Methods

### Participants

Recruitment for the study was achieved via advertisement among university students and by word of mouth. From an original opportunity sample of 110 participants, 52 people were excluded from the study on the basis that they either used an illicit substance (e.g., cannabis, ecstasy) or were smokers. The remaining 58 participants were young adults (aged between 18–35 years) studying at university. Twenty-five of these made up the AHG (mean age = 22.3 years, SD = 2.56) and 33 made up the NHG (mean age = 23.2 years, SD = 3.41). Participants were unpaid volunteers and were comprised of students studying at a university in the North East of England. A Chi-Square analysis revealed that there was no significant difference in terms of sex of participant between the AHG (12 males, 13 females) and the NHG (13 females, 20 males) groups (χ^2^ = 0.43, *df* = 1, *p* = 0.51).

### Design

The study adopted a between-subjects design comparing the AHG and NHG on PM. The main dependent measure was the score on the PRVP. Age, the number of alcohol units consumed per week, the number of years spent drinking, as well as anxiety and depression scores were included as covariates. The order of presentation of the test materials remained constant across the participants.

### Materials

#### Objective Prospective Memory (PM)

PM was assessed using the PRVP-which is a laboratory-based measure of PM based on a methodology used by earlier researchers to study the deleterious impact of cannabis upon PM in adults and PM deficits associated with binge drinking in older teenagers (Bartholomew et al., 2010; Heffernan et al., 2010). During this procedure, the participant was required to remember a series of 12 location-action combinations, which would later feature on a 10-min CD clip of a shopping high street viewed by the participant. Examples of these location-action combinations include “When you reach a shop called the Card Store” (location), “Ask directions to the train station” (action) and “When you reach the store called Clinton Cards” (location), “Note what event is highlighted in the shop window” (action). The district displayed on the CD clip is one of a busy shopping area comprising many retail shops, with many shop fronts and signs, as well as passers-by. The participant was instructed to only write down each location-action combination on a response sheet when the familiar location was reached on viewing the CD clip and not before, to ensure that each combination was recalled as part of the ongoing PM task presented on the CD clip. One point was scored for each successful location-action combination recalled, with a maximum of 12 points achievable and with the higher the score indicating a more proficient PM. In order to increase the ecological validity of the task and to make it more akin to real life, the participant was instructed to engage in an ongoing task in addition to the main PM task. This involved the participant noting on the bottom of the response sheet how many people accompanied a pushchair or pram that passed by the camera, of which there were several. This ongoing task was not assessed or analyzed, and was merely included to provide a dual task paradigm, which increased the ecological validity of the task.

#### Alcohol and Other Substance Use

Alcohol and other substance use were assessed using a modified version of the University East London Recreational Drug Use Questionnaire (RDUQ) used in previous research (Bartholomew et al., 2010; Heffernan et al., 2010; Parrott et al., 2002). The RDUQ measured alcohol use in terms of the number of units consumed per week (the following UK guidelines were provided: 1 unit of alcohol equates to 1 = 2 pint of normal strength beer, a standard 125 ml glass of wine or 1 × 25 ml measure of spirit; with each unit being equal to approximately 8 g or 10 ml of pure alcohol), their last alcohol use in hours and how many years they had been drinking. Similar details of other drug use (e.g., smoking, ecstasy, cannabis, etc.) was also collected.

#### Anxiety and Depression

Anxiety and depression indices were measured using the Hospital Anxiety and Depression Scale (HADS; Zigmond and Snaith, 1983) which is a 14-item self-report standardized questionnaire which was developed to identify varying states of depression and anxiety in non-psychiatric samples (Crawford et al., 2001). Seven items relate to general anxiety symptoms; for example; “I feel tense or “wound up” and seven items relate to generalized depressive symptoms; for example; “I feel as if I am slowed down”. Each response was scored on a four-point rating scale from zero (indicating little or no symptom) to three (indicating that the person exhibited a great deal of that particular symptom). Separate scores were calculated for anxiety and depression, with the scoring ranging from 0 to 21 for each construct (anxiety and depression) and with the higher the score the greater the severity of generalized anxiety/depression felt by the person.

#### Alcohol Hangover Symptoms

The Acute Hangover Scale (Rohsenow et al., 2007) measured the number and severity of the AH symptoms experienced. This is a self-report measure of nine symptoms typically accompanying the AH; “Hangover”, Thirsty, Tired, Headache, Dizziness, Loss of Appetite, Gastro-Intestinal Problems, ranging from zero symptoms to nine symptoms reported. Three or more symptoms was taken here to indicate the presence/absence of a set of AH symptoms and a mean score of AH symptoms was computed for each participant. The severity of these symptoms was also calculated; with severity being rated from zero (none) to seven (where the severity was “incapacitating”), with an average severity rating calculated (across the number of symptoms reported) for each participant.

#### Breath Analyzer

The Draeger AlcoDigital 3000 digital breath analyzer was used to measure the alcohol content of each participant’s breath in order to confirm that each participant’s blood alcohol concentration had returned to zero.

#### Procedure

The study was approved by the Life Sciences Ethics Committee at Northumbria University and was in accordance with the British Psychological Society Code of Ethic. All participants were adults over the age of 18 years and provided written consent for their participation. No vulnerable adults took part in the study. All participants were informed of what procedures they had to complete and were debriefed following their participation. They were also given the right to withdraw their data after taking part in the study or anytime afterwards up until the study has been published. The state of AH was produced during a natural night out of the participant’s usual drinking and each participant was tested the following day. Each participant was told that the study would explore the relationship between substance use, general feelings of anxiety and depression and everyday memory, not that the main focus of the study was to investigate the impact of the AH state upon PM, therefore some minor deception was used in order to avoid any expectancy effects. However, following each participant’s testing, he/she was fully debriefed and informed of their right to withdraw their data; none chose to do so. Testing took place on an individual basis in a quiet and undisturbed laboratory setting. Each participant was presented with the PRVP task first, followed by the substance use questionnaire, the HADS and the acute AH scale, and finally they were asked to complete the alcohol breath test. The procedure took approximately 30 min to complete. Each participant was thanked for their co-operation and provided with details of how they could withdraw their data if they so wished.

#### Analysis

Two independent *t*-tests were applied to the data in order to confirm whether the AHG differed from the NHG in terms of the number of AH symptoms experienced and the severity of these symptoms. A univariate ANCOVA was applied to the PRVP scores (controlling for age, the number alcohol units consumed per week, the number of years spent drinking, HADS anxiety and HADs depression scores) in order to compare performance on the main PRVP task between the AHG and NHG.

## Results

Table 1 contains the means and standard deviations (in brackets) for age, alcohol indices (the number of units consumed per week, the number of years spent drinking), HADS depression and HADS anxiety scores, the number of AH symptoms and severity, and scores on the PRVP, comparing the AHG and NHG.

**Table 1:** Means (and standard deviations) comparing the AHG and NHG on age, the number of alcohol units consumed per week, the number of years spent drinking, hospital anxiety and depression scale (HADS) depression and HADS anxiety scores, the number of AHG symptoms and severity of these symptoms, and scores on the Prospective Video Remembering Task (PRVP).

	Alcohol Hangover Group (AHG; *n* = 25)	Non Hangover Group (NHG; *n* = 33)
Age	22.3 (2.56)	23.2 (3.08)
Alcohol units per week	25.9 (5.01)	16.4 (6.76)
Years drinking alcohol	5.20 (2.19)	6.03 (2.93)
HADS Depression	3.04 (1.85)	3.00 (2.97)
HADS Anxiety	3.92 (2.43)	5.12 (3.69)
Hangover Symptoms	6.88 (0.92)	1.57 (0.90)
Hangover Severity	3.25 (1.01)	0.48 (0.32)
PRVP scores	5.16 (2.24)	7.51 (2.12)*

*Significant at the P < 0.001 level.

An independent samples *t*-test revealed significantly more AH symptoms reported in the AHG compared with the NHG (*t*_(56)_ = 9.14, *p* < 0.01; 6.88 vs. 1.57 for AHG and NHG respectively). An independent samples *t*-test revealed significantly greater severity scores reported in the AHG compared with the NHG (*t*_(56)_ = 14.7, *p* < 0.01; 3.25 vs. 0.48 for AHG and NHG respectively). A univariate analysis of covariance (ANCOVA) applied to the PRVP scores (controlling for age, the number alcohol units consumed per week, the number of years spent drinking, HADS anxiety and HADS depression scores) revealed significantly fewer items remembered on the PRVP by the AHG when compared with the NHG (*F*_(1,51)_ = 5.69, *p* < 0.05; 5.16 vs. 7.51 for AHG and NHG respectively).

## Discussion

Previous research has shown that the AH has a detrimental effect upon cognitive abilities, including psychomotor performance, memory and attentional deficits, yet no published research articles to date have focused on what impact the AH might have upon everyday memory. The current study focused upon PM (remembering future actions, such as remembering to meet with a friend at a future point in time, or remembering to take an important medication on time; Brandimonte et al., 1996), which provides a good example of everyday memory. For this purpose, the current study compared an AHG with a NHG on an objective measure of PM in the form of the PRVP. The results demonstrated that, after controlling for variations in age, the number of units of alcohol consumed per week, the number of years spent drinking, as well as anxiety and depression indices, being in a state of AH had a significant detrimental effect on PM performance. This findings from the present study can be added to the growing list of cognitive and memory deficits known to be adversely affected by a state of AH, including attentional and memory deficits, problems with information processing and short-term memory deficits, as well as deficits in working memory and inhibition tasks (Kim et al., 2003; Howland et al., 2010; Rohsenow et al., 2010; McKinney et al., 2012; Owen et al., 2013; Grange et al., 2016). This study is important as it contributes to a limited number of studies investigating the impact a state of AH has upon cognition and memory. It is worth noting that, in the present study, the ongoing task and the main PM task may differ in terms of their cognitive demands. The ongoing task utilized in the present study may rely more on attentional monitoring (when a pushchair passes by, count the number of people accompanying it), whereas the main PM task requires more demanding cognitive processes that not only require the recognition of a cue in the environment (a shop front or specific person who appears) and remembering to carry out a specific activity when this “cue” appears. Therefore, future research may wish to adopt a more theoretically driven methodology, such as the use of the multi-process framework (McDaniel et al., 2004) as framework. PM is a form of memory that is constantly used within everyday life and enables us to carry out intended actions; these range from simple tasks, such as remembering lock one’s door upon leaving the house, to the fairly complex, such as remembering organize a birthday party for a close relative or carrying out a set important procedures within the workplace. Therefore, good PM functioning is seen as pivotal to proficient human functioning and it is therefore no surprise that PM deficits are the most frequently reported everyday memory failures in life (see e.g., Kliegel and Martin, 2003). Given that good PM function plays an important role in all walks of life, including healthy adolescent development (Geurten et al., 2016), efficient performance in the workplace (Finstad et al., 2006) and healthy ageing (Parikh et al., 2015), then impairments or reduced functioning as a result of a state of AH can significantly compromise PM performance within these domains.

Although the present study identified significant deficits in the PRVP, it is clear that the PM items contained in the PRVP are event-based items, that is when a specific cue appears in the environment (on the CD clip of the busy high street) than this may trigger the PM action that is to be remembered. More recent thinking on PM (Kliegel et al., 2008) distinguishes between both event-based and time-based PM (time-based PM is a scenario where the to-be-remembered PM action is purely based on the participant’s self-monitoring of the time and he/she remembering to complete a specific action following that period of time). Future research should include both time- and event-based PM actions to observe whether there are selective or global impairments in PM associated with being in a state of AH (see e.g., the “virtual office” environment created by Jansaari et al., 2012). Indeed, a more comprehensive battery of memory assessments, including a series of PM tests, would provide a fuller picture of the range of memory deficits associated with the AH, many of which would interact with PM, such as episodic memory and executive function. Biological drug-screening methods would provide a more accurate view of other drug-use habits, rather than relying on self-reported measures (Babor and Kadden, 2005). Although the digital breathalyzer was used to ensure that the BAC for each participant had returned to zero prior to testing, future research might wish to collect data in order to calculate eBAC readings for each participant/group. Although the use of a between-subjects design was appropriate for the current study, it does have the limitation that it is impossible to control for all differences between the two groups, such as general life-style differences, diet, etc. Future research should also include a non-alcoholic group so that PM performance in the two alcoholic groups can be compared with a non-drinking control group. This would enable us to determine whether the NHG has a sufficiently intact PM capacity similar to that of non-drinking controls. Since heavy episodic drinking can begin as early as 12–14 years of age (Siqueira and Smith, 2015), future research should examine what impact a state of AH (or repeated states of AH) might have upon a child’s neurocognitive development, given that the brain is still developing across this critical period of time (Brown et al., 2000; Spear, 2013, 2015; Fleming, 2015). Any injurious impact upon this development because of a state of AH could interfere with related cognitive processes, including everyday PM, producing long-lasting deficits to brain structure and function.

## Conclusion

The present study has demonstrated that everyday PM deficits are associated with a state of AH, and that this finding cannot be attributed to differences in age, anxiety or depression indices, or alcohol use between the AHG and NHG. PM deficits should be added to the growing list of cognitive sequelae associated with a state of AH. Such findings may prove useful in educating young people as to the adverse cognitive consequences of being in a state of AH.

## Author Contributions

TH designed the experiment, conducted the required data analysis, wrote the draft version and final version of the manuscript.

## Conflict of Interest Statement

The author declares that the research was conducted in the absence of any commercial or financial relationships that could be construed as a potential conflict of interest.
